# Formulation of a Model Resin System for Benchmarking Processing-Property Relationships in High-Performance Photo 3D Printing Applications

**DOI:** 10.3390/ma13184109

**Published:** 2020-09-16

**Authors:** Jianwei Tu, Kamran Makarian, Nicolas J. Alvarez, Giuseppe R. Palmese

**Affiliations:** Department of Chemical and Biological Engineering, Drexel University, Philadelphia, PA 19104, USA; jt3229@drexel.edu (J.T.); km3778@drexel.edu (K.M.); nja49@drexel.edu (N.J.A.)

**Keywords:** 3D Printing, resin formulation, photo-polymerizable resins

## Abstract

A well-defined resin system is needed to serve as a benchmark for 3D printing of high-performance composites. This work describes the design and characterization of such a system that takes into account processability and performance considerations. The Grunberg–Nissan model for resin viscosity and the Fox equation for polymer *T_g_* were used to determine proper monomer ratios. The target viscosity of the resin was below 500 cP, and the target final *T_g_* of the cured polymer was 150 °C based on tan-δ peak from dynamic mechanical analysis. A tri-component model resin system, termed DA-2 resin, was determined and fully characterized. The printed polymer exhibited good thermal properties and high mechanical strength after post-cure, but has a comparatively low fracture toughness. The model resin will be used in additive manufacturing of fiber reinforced composite materials as well as for understanding the fundamental processing–property relationships in light-based 3D printing.

## 1. Introduction

Three-dimensional (3D) printing is an additive manufacturing process in which successive layers of material are patterned and combined to form 3D shapes. 3D printing technologies are currently experiencing financial growth and are being increasingly adopted across industries. Factors driving this market growth are aggressive research and development and the growing demand for prototyping applications from industries such as healthcare, automotive, defense, and aerospace [1]. In fact, the aerospace 3D printing market was estimated to be USD 1.86 billion as of 2019—only 16.8% of the total 3D printing market—and is expected to grow annually at a rate of 16.9% over the next 7 years to reach USD 6.72 billion in 2027 [2]. For 3D patterning polymeric materials, extrusion or melt type techniques, such as fused deposition modelling and selective laser sintering, are common methods for the fabrication of thermoplastic parts. However, these techniques have the drawback of comparatively low resolution, weak layer adhesion, and slow processing. On the other hand, in light-based methods, the printing resolution and production speed are drastically improved due to the exceptional spatial control and versatility of photo-polymerization reactions [3]. Moreover, the mechanical properties of the printed objects are significantly enhanced due to better layer-to-layer cohesion. Thus, light-based technologies offer attractive routes for 3D printing of polymers and composites. Examples of technologies include stereolithography (SLA), digital light processing (DLP), and continuous liquid interface production (CLIP) [4,5,6]. In SLA, specific surface regions of photo-sensitive liquid resin undergo localized polymerization by exposure to a scanning spot light source. In DLP, all given portions of a layer are simultaneously photocured, significantly reducing part production times. The recently developed CLIP process utilizes a continuous building strategy, which further increases part production speed and enhances surface finish.

Light-based methods use photo-sensitive resins that can be cured by a light source, often a UV laser. Typical materials used are acrylic and epoxy resins. Currently, most standard photo-polymerizable resin formulations on the market produce parts with relatively low thermal and mechanical properties and, therefore, cannot be used for additive manufacturing of high-performance composite materials. Moreover, the compositions of commercial resins are proprietary information in most formulations. A list of commercial resins from reputable suppliers and their property information can be found in Table A1 in Appendix A. To develop a fundamental understanding of the processing-property relationships underlying light-based 3D printing methods, a well-defined additive manufacturing resin formulation is needed to serve as the benchmark resin system. A standard resin formulation should have the following characteristics [7]: (i) commercially available component monomers; (ii) good storage stability including long resin shelf life and low monomer volatility; (iii) low viscosity for facile printing; (iv) good final part properties including good dimensional stability and high thermal and mechanical properties.

Bisphenol A glycerolate dimethacrylate (Bis-GMA), also known as the vinyl ester of diglycidyl ether of bisphenol A (VE-DGEBA), is a major component commonly used in dental formulations and in vinyl ester resins [8,9,10]. Due to the presence of rigid bisphenol-A core in the backbones, the molecule imparts excellent performance characteristics to its final products. The strong intermolecular interactions by hydroxyl groups, however, result in an extremely viscous resin at room temperature [11], so the use of diluent co-monomers becomes necessary for easy handling [12]. Styrene is the most common comonomer in vinyl ester resins, but it cannot be used for 3D printing because it is a hazardous air pollutant (HAP) and a volatile organic compound (VOC, vapor pressure at 25 °C is 6.5 mmHg) [10,13,14]. On the other hand, triethylene glycol dimethacrylate (TEGDMA) has been widely used as a comonomer of Bis-GMA in dental formulations. It significantly reduces the viscosity of the mixture and increases the polymer degree of conversion [15]. However, the addition of TEGDMA causes an undesirable increase in polymerization shrinkage due to its higher double bond concentration and increased overall double bond conversion [16,17]. Neat TEGMDA can shrink by 12.3% compared to 5.2% shrinkage of Bis-GMA after polymerization [18]. For this reason, low viscosity monomers with higher molecular weights were developed to decrease polymerization shrinkage and improve processability. To this end, ethoxylated bisphenol A dimethacrylate (Bis-EMA) has been present in several commercial formulations, partially or totally replacing TEGDMA [19,20]. The molecular structure of Bis-EMA monomer is almost the same as Bis-GMA monomer, except for the absence of hydroxyl groups. It shows intrinsically low viscosity due to the absence of hydroxyl groups that form hydrogen bonding. The lack of hydroxyl groups also results in a more hydrophobic molecule, which makes Bis-EMA suitable for applications where moisture uptake is undesirable. The equilibrium water uptake of neat Bis-EMA polymer is only around 0.6~1.8%, as compared to 2.5~3.1% for Bis-GMA polymer and 6.0~6.3% for TEGDMA polymer [18,21,22].

This work describes the design of a well-defined benchmark resin formulation that in the future will be used to investigate the processing-property relationships in 3D printing. The screening process for resin development was primarily based on viscosity and glass transition temperature (*T_g_*) considerations. Predictive models, namely the Grunberg–Nissan model for resin viscosity and the Fox equation for polymer *T_g_*, were used as guiding tools to determine proper monomer ratios. The preference of di-functional reactive diluents over mono-functional is emphasized in terms of dimensional stability. The benchmark resin formulation is presented and fully characterized herein.

## 2. Materials and Methods

### 2.1. Materials

**Scheme 1:**
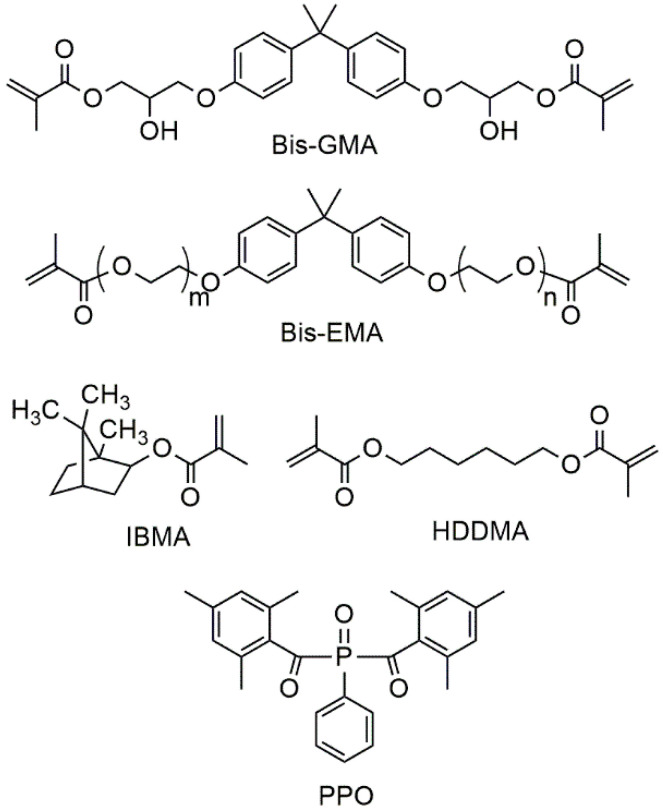
The chemical structures of the monomers and the photo-initiator.

Bisphenol A glycerolate dimethacrylate (Bis-GMA, M_w_~512 g/mol), ethoxylated bisphenol A dimethacrylate (Bis-EMA, M_w_~540 g/mol), and 1,6-hexanediol dimethacrylate (HDDMA, M_w_ = 254 g/mol) were supplied by Esstech, Inc. (Essington, PA, USA). Isobornyl methacrylate (IBMA, M_w_ = 222 g/mol) and phenylbis(2,4,6-trimethylbenzoyl)phosphine oxide (PPO, or bisacylphosphine oxides, BAPO) were purchased from MilliporeSigma (St. Louis, MO, USA). The molecular structures of the chemicals are given in Scheme 1. All chemicals were used as received.

### 2.2. Methods

#### 2.2.1. Resin Formulation and Printing

To prepare photo-polymerizable resins for DLP 3D printing, the monomers with determined weight ratios were evenly mixed, then 0.7 wt.% PPO (based on resin weight) was added as the photo-initiator to the resin. The mixture was stirred in an amber bottle to block UV light exposure until the photo-initiator completely dissolved. Upon degassing, the resin is ready for DLP printing. Samples were printed in an Anycubic Photon DLP printer (Shenzhen, China). The wavelength of the projected light by the printer is 405 nm. The light intensity is 0.45 ± 0.05 mW/cm^2^ determined by a radiometer (ILT2400, International Light Technologies, Peabody, MA, USA). The default print settings for this study were 100 μm layer thickness and 100 s exposure time and 10 s off time between layers. As-printed “green” parts were subject to the following post-processing procedure: samples first undergo photo post-cure in a blue light oven (Form Cure, Formlabs Inc., Somerville, MA, USA) at 80 °C for 2 h, followed by thermal post-cure in a conventional laboratory oven at 120 °C for 3 h, ramping to 180 °C in 60 min and isotherm at 180 °C for 30 min.

#### 2.2.2. Working Curve

To create the working curve, several samples were 3D printed at different values of exposure time to achieve different thicknesses (cure depth). This requires the build platform to be removed from the vat to allow samples to have a variety of thicknesses. Initially, three coin-shaped samples with the approximate diameter of 2 cm were printed simultaneously at a specific exposure time and as a single print layer. Next, their thicknesses were measured using a ratchet micrometer and averaged to provide the average cure depth for that exposure time. This process was repeated for different exposure time durations to obtain different values of cure depth for each case. Finally, the working curve was constructed by plotting the average cure depth values against their corresponding energy doses on a logarithmic axis. Note that for each run, the energy dose is calculated by multiplying the exposure time of that run by the printer’s light intensity. The slope and the x-intercept of the logarithmic curve fit are the resin properties known as depth of penetration (*D*_p_) and critical energy dose (*E*_c_) [23].

#### 2.2.3. Characterization

Steady shear viscosity measurements were performed on TA Instruments AR2000 rheometer (New Castle, DE, USA) at 25 °C under steady state shear mode using a cone-plate geometry with shear rate ramping from 0.001 s^−1^ to 100 s^−1^. For every resin, three viscosity measurements were taken. Fourier transform near infrared (FT-NIR) experiments were performed on a Nicolet iS50 FT-IR spectrometer (Thermo Fisher Scientific, Waltham, MA, USA), operating in transmission mode with a deuterated triglycine sulfate (DTGS) detector. FT-NIR spectra were recorded with 32 scans at a 4 cm^−1^ resolution in 4000–8000 cm^−1^ range. Dynamic mechanical analysis (DMA) experiments were performed on bar samples of size 35 mm × 12.7 mm × 3.2 mm using a TA Instruments Q800 Dynamic Mechanical Analyzer in a single cantilever mode with the oscillation frequency set to 1 Hz, the amplitude set to 10 µm, and temperature ramping at a rate of 2 °C/min. Resin densities were measured using an Anton Paar DMA 500 density meter (Graz, Austria), and a density value was obtained as an average over three measurements. Densities of cured polymers were determined using a density-gradient column in accordance with ASTM D1505-18 standard [24]. For each polymer, at least three samples were tested. The liquid system in the column consisted of water and sodium bromide. All mechanical testing was performed on an Instron tester, model #: A1740-3003 (Norwood, MA, USA). Tensile testing was carried out according to ASTM D638-14 standard [25]. At least five dog-bone specimens of type IV were tested at a test speed of 5 mm/min. An extensometer was applied to obtain accurate strain values, and the tensile modulus was calculated based on data up to 0.25% strain. Flexural properties were determined according to ASTM D790-17 standard [26]. At least three long rectangular bars of size 120 mm × 12.7 mm × 3.2 mm were tested in a three-point bending configuration and the span-to-depth ratio was kept at 16. Fracture toughness was measured using at least five single-edge-notch-bend (SENB) specimens according to ASTM D5045-14 standard [27]. Pre-cracks were initiated by tapping a fresh razor blade inserted in the notch.

## 3. Results and Discussion

In many resin formulations, the preferred major component is Bis-GMA resin because of its good final properties. The addition of Bis-EMA resin maintains a low cure shrinkage, but also reduces resin viscosity and final material moisture sensitivity significantly. In this work, a combination of Bis-GMA and Bis-EMA served as the base resin of the formulations. A reactive diluent is needed to further reduce the viscosity to make suitable formulations for DLP printing. Two reactive diluents, isobornyl methacrylate (IBMA) and 1,6-hexanediol dimethacrylate (HDDMA), were selected for the study after screening. HDDMA had been used in this research group as a reactive diluent in vinyl-ester resins [13]. HDDMA is known for its hydrophobic nature. A photo-cured polymer network containing HDDMA was found to uptake three to four times less water compared to the corresponding network containing the same amount of TEGDMA [28]. IBMA was selected for the high *T_g_* of its polymer [29,30,31,32]. Both diluents have high boiling points (258 °C for IBMA and 315 °C for HDDMA) and low vapor pressures (0.01 mmHg at 25 °C for IBMA and 0.02 mmHg at 100 °C for HDDMA) [33,34]. The use of monomers with low vapor pressures is essential for a safe work environment and minimizes the composition drift due to possible monomer evaporation during printings. Table 1 summarizes the measured room temperature viscosities of the component monomers, and the *T_g_*s of corresponding neat polymers available from the literature.

Predictive models for resin viscosity and polymer *T_g_* were used as guiding tools to determine proper monomer ratios. The Fox equation, shown in Equation (1), has been widely used to predict the *T_g_* of a polymer mixture based on the *T_g_*s of the neat components [42]:

(1) 1Tg=∑ωiTg,i,

where *T_g,i_* and *ω_i_* are the glass transition temperature and the mass fraction of component *i*, respectively. The simplest model for predicting the viscosity of liquid mixtures is the Arrhenius equation [43], but the additive model neglects thermodynamic parameters characteristic of the interactions between components and results in inaccurate predictions. The Grunberg–Nissan model, based on a modification of the Arrhenius equation to account for the excess free energy of mixing, shown in Equation (2), is commonly used to describe the viscosity of liquid mixtures [44,45,46]:

(2) $$\ln\eta =\sum_{i}x_{i}\ln\eta_{i}+\sum_{i}\sum_{j}x_{i}x_{j}G_{ij},$$

where *η* is the viscosity of a mixture, *η_i_* and *x_i_* are the viscosity and the mole fraction of each component in the mixture, respectively, and *G_ij_* is an interaction parameter dependent on the components and temperature. A negative value of *G_ij_* indicates favorable mixing.

Binary interaction parameters *G_ij_* were determined from the viscosity measurements of binary liquid mixtures of the monomers and calculated using Equation (2). The interaction parameters are found to be relatively constant with regards to monomer mixing ratios (see Table A2). Table 2 lists the averaged interaction parameters between Bis-GMA and Bis-EMA; Bis-GMA and a reactive diluent; and Bis-EMA and a reactive diluent such as *G*_12_, *G*_13_, and *G*_23_, respectively. The interaction parameters show that Bis-GMA mixed favorably with Bis-EMA and HDDMA, but IBMA did not efficiently mix with Bis-GMA or Bis-EMA, likely due to the bulky nature of the isobornyl group that hinders flow [47]. Interestingly, the excess free energy of mixing is close to zero when mixing Bis-EMA with HDDMA.

**Figure 1:**
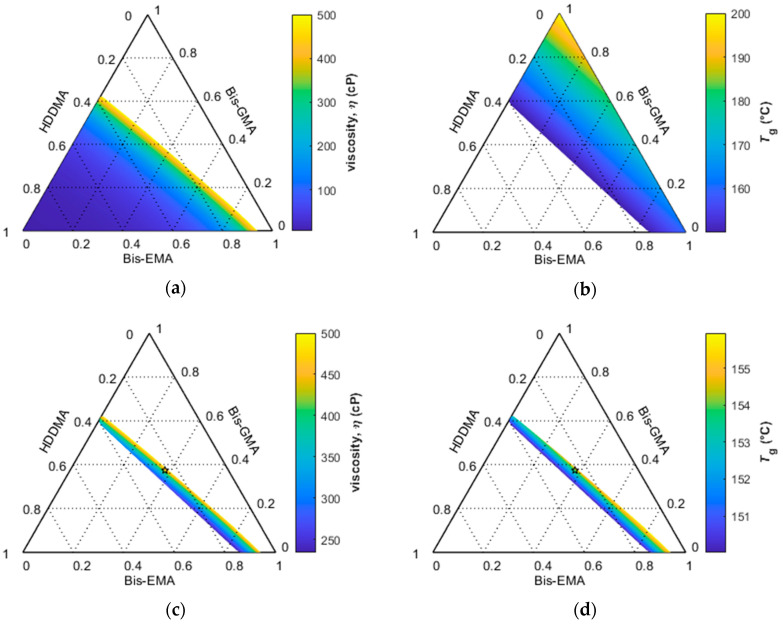
Ternary plots of predicted viscosity and *T_g_* as functions of changing composition of Bis-GMA/Bis-EMA/HDDMA system; the axes are weight percentages, and values are represented by color. (**a**) Predicted viscosity lower than 500 cP; (**b**) predicted *T_g_* higher than 150 °C; (**c**,**d**) predicted overlap area where viscosity is lower than 500 cP and *T_g_* is higher than 150 °C. The star in (**c**,**d**) marks the DA-2 composition.

**Figure A1:**
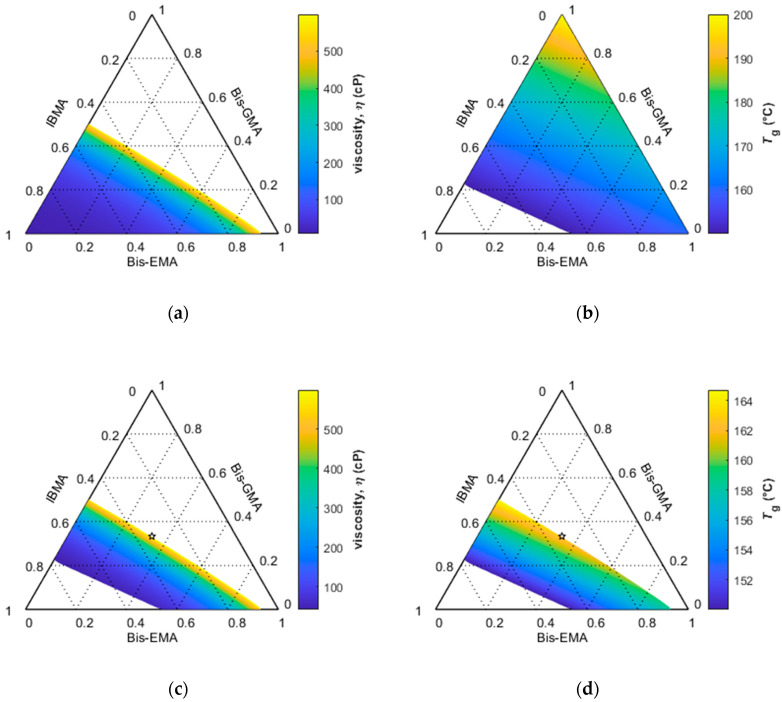
Ternary plots of predicted viscosity and *T_g_* as functions of changing composition of Bis-GMA/Bis-EMA/IBMA system; the axes are weight percentages, and values are represented by color. (**a**) Predicted viscosity lower than 600 cP; (**b**) predicted *T_g_* higher than 150 °C; (**c**,**d**) predicted overlap area where viscosity is lower than 600 cP and *T_g_* is higher than 150 °C. The star in (**c**,**d**) marks the DA-1 composition.

Figure 1a,b, shows the predictions of viscosity and the *T_g_* of final cured material using the Grunberg–Nissan model and the Fox equation, respectively, for ternary mixture system Bis-GMA/Bis-EMA/HDDMA. In Figure 1a, the shaded area denotes a predicted viscosity ≤500 cP. In Figure 1b, the shaded area denotes a predicted final *T_g_* higher than 150 °C. Figure 1c,d show the overlap area where the predicted viscosity is lower than 500 cP and *T_g_* is higher than 150 °C. The same graphs for Bis-GMA/Bis-EMA/IBMA system are shown in Figure A1 (Appendix C). Note the slightly higher cut-off viscosity, 600 cP. For the *T_g_* predictions, the final *T_g_*s of Bis-GMA, BisEMA, and IBMA polymers are 200 °C, 160 °C, and 140 °C, respectively [29,30,31,32,35,36,37,38,39]. The *T_g_* of the HDDMA polymer is not commonly reported in the literature, but it should be higher than the *T_g_* of its acrylate counterpart, 1,6-hexanediol diacrylate (HDDA), which was reported to be 93 °C [29,40]. Only one reference [41] reported 150 °C *T_g_* for the HDDMA polymer. To be conservative, the final *T_g_* of HDDMA polymer is assumed to be 110 °C for predictive calculations. *T_g_* is a primary consideration only once the viscosity values are satisfactory for facile 3D printing. In the predicted overlap areas shown in Figure 1c,d, higher *T_g_*s appears on the high viscosity side. Additionally, other factors need to be considered to obtain optimal material properties. Bis-EMA polymer has a relatively low Young’s modulus, typically less than 2 GPa [22,48]. Also, impact resistance was reported to decrease as Bis-EMA was added to the Bis-GMA/Bis-EMA copolymer [49]. This can be explained by the decrease in the overall strength of intermolecular interactions; as the hydroxyl group concentration decreases, the number of physical crosslinking sites is reduced. On the other hand, if the Bis-GMA content is increased, more reactive diluent is needed, which will increase cure shrinkage. Therefore, to retain mechanical performance and minimize cure shrinkage, the formulations at the center of the high viscosity side of the overlap areas were selected for this study. These two formulations are called DA-1 and DA-2. Given in Table 3, DA-1 consists of Bis-GMA 33.3 wt.%, Bis-EMA 33.3 wt.%, and IBMA 33.3 wt.%, and DA-2 consists of Bis-GMA 37.5 wt.%, Bis-EMA 37.5 wt.%, and HDDMA 25 wt.%. The predicted viscosities for the DA-1 and DA-2 resins based on the Grunberg–Nissan model are 620 cP and 450 cP, respectively. Rheological measurements showed DA-1 and DA-2 have viscosity values of 580 ± 40 cP and 490 ± 50 cP, respectively. The values are summarized in Table 4.

**Figure 2:**
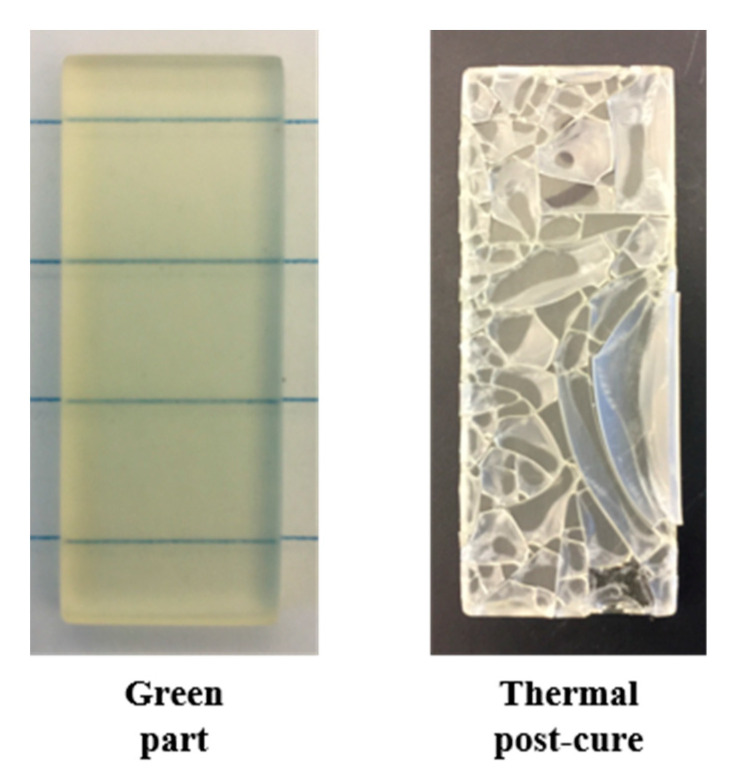
Cracks developed during thermal post-cure of a DA-1 green part.

DA-1 and DA-2 green parts were printed using the default print settings (100 μm layer thickness and 100 s exposure time). Green parts created by room temperature DLP printing often need to be post-cured to promote additional conversion. The post-cure process is especially necessary for parts printed with high *T_g_* resins because these resins reach vitrification at low monomer conversions under the printing temperature [50]. When a DA-1 green part was directly placed in a conventional oven for thermal post-cure, cracks developed throughout the part (shown in the right picture of Figure 2) and some delamination was observed between layers. This is due to the unreacted monofunctional isobornyl methacrylate molecules, which diffuse out before they can react because the thermal activation of the methacrylate double bond reaction is relatively slow [47]. Such cracking and delamination phenomena would not happen if mono-functional monomers of higher reactivity were used, such as *p*-methyl styrene and *N*-vinylpyrrolidone. Another way to overcome this problem is to use multifunctional (≥2) monomers, proved by the case of DA-2. Direct thermal post-cure of DA-2 green parts did not cause cracks or delamination because most unreacted reactive diluent functionality exist as dangling chain ends. For this reason, the DA-2 formulation was chosen as the standard resin for the study.

**Figure 3:**
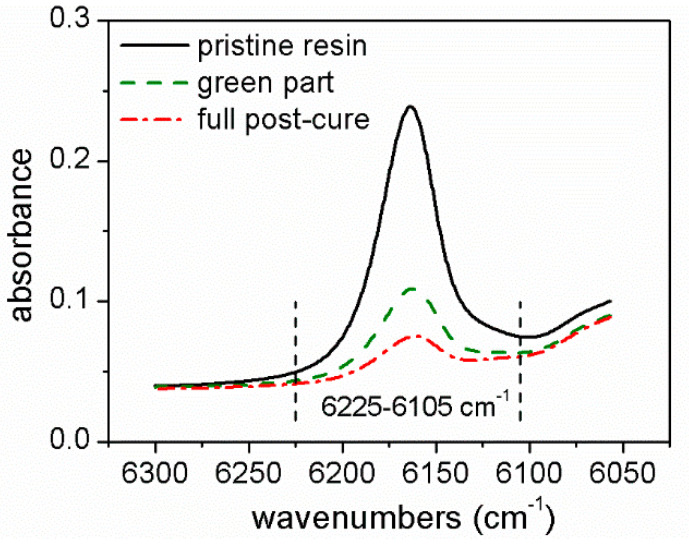
FT-NIR absorption band around 6225–6105 cm^−1^ for DA-2 conversion calculation.

**Figure 4:**
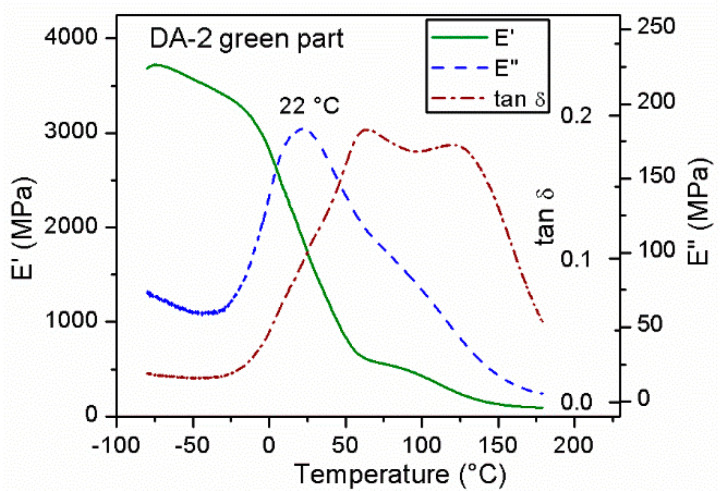
DMA thermogram of a DA-2 green part.

**Figure 5:**
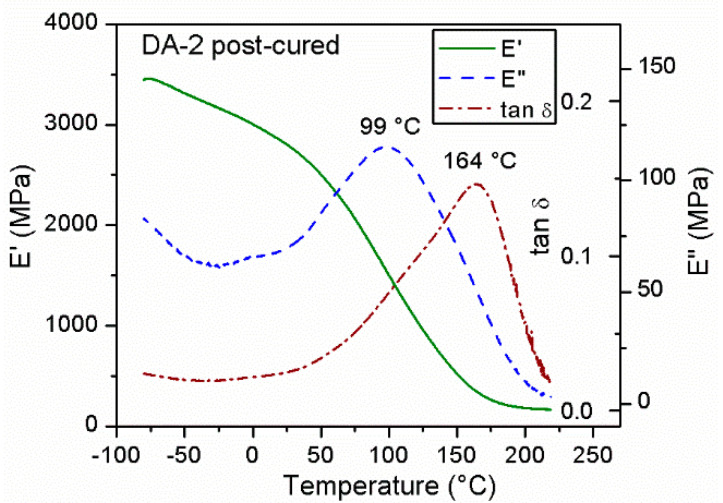
DMA thermogram of post-cured DA-2.

The fractional monomer conversion, *α*, is determined using Equation (3) from the decreasing integral of the characteristic near-IR absorption band of the methacrylate double bond, shown in Figure 3 [51]:

(3) $$\alpha =1-\frac{A_{1}\left(6225-6105\;cm^{-1}\right)}{A_{0}\left(6225-6105\;cm^{-1}\right)},$$

where *A*_0_ and *A*_1_ are the area integrals of the absorption band from 6225–6105 cm^−1^ of the pristine resin and cured sample, respectively. Note that the calculated conversion is based on the absorbance averaged over the thickness of the tested sample. The fractional conversion is 0.67 for a DA-2 green part. Figure 4 shows the DMA thermogram of a DA-2 green part with a *T_g_* of 22 °C identified as the peak of loss modulus, *E*″. The *T_g_* value indicates that the vitrification conversion of the DA-2 resin is around 0.67 at room temperature. The tan δ curve shows a bimodal glass transition with a slightly more intense peak at 64 °C and a very broad second one at 120 °C. The *E*″ curve also shows a shoulder above the main transition. Though this might be interpreted as a heterogeneous network morphology or spatial inhomogeneity of reaction, the likely cause of the behavior is a dark reaction by trapped radicals upon heating past the first *T_g_* resulting in partial revitrification and devitrification upon continued heating. The slight plateau in *E*′ following the first transition supports this explanation. After post-processing, the fractional conversion becomes 0.88. Figure 5 shows the DMA thermogram of a fully post-cured DA-2 sample. Based on three DMA tests, the *T_g_* of post-cured DA-2 material is 97 ± 3 °C and 165 ± 4 °C, identified as the maxima of loss modulus *E*″ and of tan *δ*, respectively. The latter number agrees with the predicted 160 °C *T_g_* via the Fox equation.

**Figure 6:**
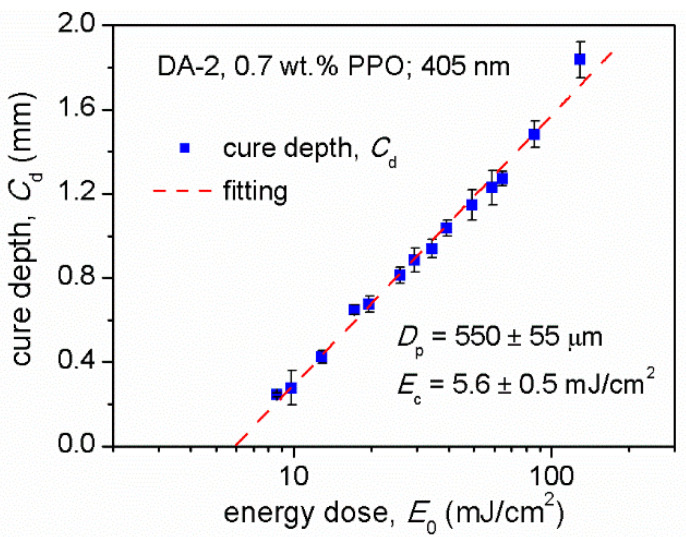
The working curve of the DA-2 resin.

Successful printing of objects by stereolithography requires predetermined knowledge of the photo-curing properties of the starting material. Principles laid out by Jacobs to describe the photo-polymerization process were used to create a working curve that provides two key parameters that govern the polymerization of a photo-sensitive resin: depth of penetration, *D_p_*_,_ and critical energy of polymerization, *E_c_* [52]. Knowing *D_p_* and *E_c_* allows users to choose the appropriate settings for light exposure and z-axis increments, which optimizes the curing conditions to achieve the desired results. Here, the working curve is constructed for the DA-2 resin with 0.7 wt.% PPO photo-initiator, shown in Figure 6. Under irradiation wavelength of 405 nm, the depth of penetration *D*_p_ is 550 ± 55 µm and the critical energy *E_c_* is 5.6 ± 0.5 mJ/cm^2^. The DA-2 resin has a large *D_p_*, which enables it to 3D print fiber-reinforced composite structures. The depth of penetration can be reduced by adding photo absorbers to allow for a better printing resolution.

**Scheme 2:**
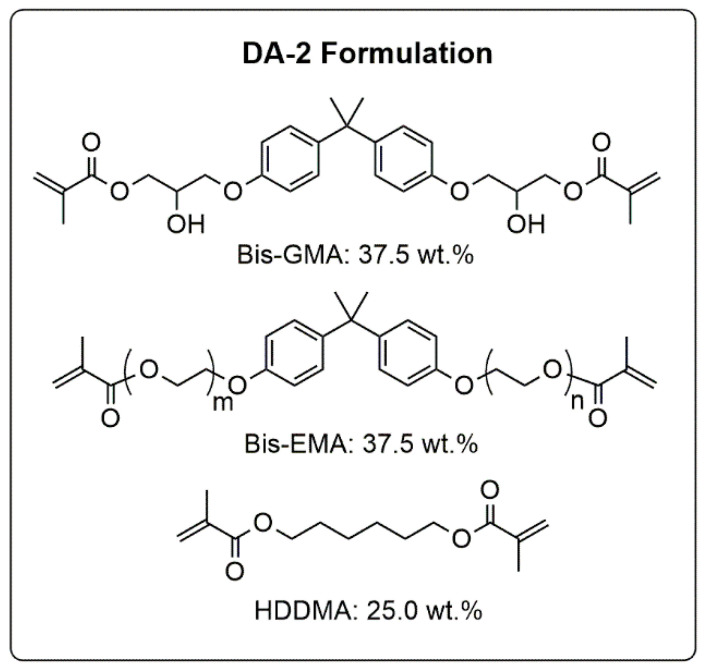
The DA-2 Formulation.

The DA-2 resin’s density is 1.105 ± 0.001 g/cm^3^, and the cured DA-2 material (fractional conversion: 0.88) has a density of 1.200 ± 0.002 g/cm^3^. Cure shrinkage is, therefore, calculated based on the shrinkage in specific volume to be 7.9%. The tensile, flexural, and fracture toughness properties are measured for the fully cured DA-2 material. Table 5 and Scheme 2 summarize the properties and the composition information of the DA-2 material. Compared to the commercial resins shown in Table A1, DA-2 is a strong material with an elastic modulus around 3 GPa and a flexural strength over 100 MPa. It has a low viscosity and a high depth of penetration for blue light curing, both of which make the resin suitable for the additive manufacturing of fiber-reinforced composites. DA-2 has a comparatively low fracture toughness that is typical of free radical polymerization systems. Future work will focus on improving the fracture toughness of the resin, studying the effects of 3D printing parameters, as well as understanding the processing-property relationships in light-based 3D printing technologies.

## 4. Conclusions

The additive manufacturing field lacks a well-defined photo-sensitive resin formulation for high performance composite applications. Current commercial resins produce parts with relatively low thermal and mechanical properties and contain proprietary composition information. The DA-2 formulation was herein developed as a model resin system for high performance 3D printing applications. Predictive models, namely the Grunberg–Nissan model for the prediction of resin mixture viscosity and the Fox equation for polymer *T_g_*, were successfully applied to determine proper monomer ratios to give optimal material properties. The clear resin has a large depth of penetration suitable for the additive manufacturing of fiber-reinforced composites. The depth of penetration can be reduced for 3D printing with the addition of photo absorbers. The high *T_g_* resin has to be post-cured after printing to achieve maximum cure due to early vitrification of the resin at the room printing temperature. The post-cured polymer exhibited good thermal properties and high mechanical strength but has a comparatively low fracture toughness, typically observed in free radical polymerization systems. Future work will focus on improving the fracture toughness of the photo-polymerizable resin, applying the resin to the additive manufacturing of fiber-reinforced composites and understanding the fundamental processing-property relationships underlying light-based 3D printing technologies.

## Figures and Tables

**Table 1:** Room temperature viscosities of monomers and the *T_g_*s of their respective polymers.

Monomer	*η* (cP)	*T_g_* (tan *δ*) (°C)
Bis-GMA	750,000	200–230 ^1^
Bis-EMA	900	150–180 ^2^
IBMA	8	110–150 ^3^
HDDMA	6	~100–150 ^4^

^1^ Ref. [35,36,37,38]; ^2^ Ref. [39]; ^3^ Refs. [29,30,31,32]; ^4^ Refs. [40,41].

**Table 2:** Interaction parameters determined for monomer mixtures.

Interaction Parameter	*G*_12_	*G*_13_	*G*_23_
Bis-GMA/Bis-EMA/IBMA	−2.76	3.85	2.05
Bis-GMA/Bis-EMA/HDDMA	−2.76	−3.68	−0.07

**Table 3:** The DA-1 and DA-2 formulations.

Formulation	Weight Fraction		
DA-1	**Bis-GMA**	**Bis-EMA**	**IBMA**
	0.333	0.333	0.333
DA-2	**Bis-GMA**	**Bis-EMA**	**HDDMA**
	0.375	0.375	0.25

**Table 4:** The predicted and measured viscosities at 25 °C and final *T_g_*s of the DA-1 and the DA-2 formulations.

Formulation	Viscosity, *η* (cP)		Final *T_g_* (°C)	
	Predicted	Measured	Predicted	Measured
DA-1	620	580 ± 40	165	-
DA-2	450	490 ± 50	160	165 ± 4

**Table 5:** DA-2 property table.

DA-2 Properties		
Viscosity, *η*	490 ± 50	cP
Density, resin, *ρ*_0_	1.105 ± 0.001	g/cm^3^
Density, cured, *ρ*	1.200 ± 0.002	g/cm^3^
Depth of Penetration, *D_p_* ^1^	550 ± 55	μm
Critical Energy, *E_c_* ^1^	5.6 ± 0.5	mJ/cm^2^
Glass transition, *T_g_* (*E*”)	97 ± 3	°C
Glass transition, *T_g_* (tan *δ*)	165 ± 4	°C
Tensile modulus, *E*	2.8 ± 0.1	GPa
Tensile strain at break, ε	2.5 ± 0.6	%
Tensile strength, *TS*	61.9 ± 6.3	MPa
Flexural modulus, *E*	3.0 ± 0.1	GPa
Flexural strength, *FS*	110 ± 10	MPa
Fracture toughness, *K_Ic_*	0.45 ± 0.02	MPa·m^1/2^
Fracture toughness, *G_Ic_* ^2^	58.8 ± 0.3	J/m^2^

^1^ DA-2 with 0.7 wt.% PPO; ^2^ Calculated using the equation: $G_{Ic}=\frac{\left(1-\nu^{2}\right)K_{Ic}{}^{2}}{E}$ , where *E* is the flexural modulus, *K_Ic_* is the experimental fracture toughness, and the Poisson’s ratio *ν* is assumed to be 0.35.

## Appendix A. Commercial SLA Resin Systems and Their Properties

**Table A1:** Commercial SLA resin systems and their properties.

Resin	Supplier		Composition		Price (USD/L)
Peopoly Model Resin	Peopoly, Inc. Ltd.		Proprietary		70
MakerJuice Standard Resin	MakerJuice Labs		Proprietary		60
Type D PRO UV Resin	DruckWege, GmbH		Proprietary		120
PR48-Clear Resin	CPS Polymers		Open source ^1^		120
VeroClear (RGD810) Resin	Stratasys, Ltd.		Proprietary		350
Standard Clear (FLGPCL04) Resin	Formlabs, Inc.		Proprietary		150
High Temp (FLHTAM02) Resin	Formlabs, Inc.		Proprietary		200
	***η* (cP)**	***HDT* (°C)**	***E* (GPa)**	***TS* (MPa)**	***IZOD* (J/m)**
Peopoly Model Resin	600	-	0.83	60	-
MakerJuice Standard Resin	146	-	1.03	52	-
Type D PRO UV Resin	33–57	-	1.12	35	-
PR48-Clear Resin	400	-	1.4	28	-
VeroClear (RGD810) Resin	70–75 ^2^	45–50	2–3	50–65	20–30
Standard Clear (FLGPCL04) Resin	800–900 ^3^	73	2.8	65	25
High Temp (FLHTAM02) Resin	N/A	238	2.9	51	24.2

^1^ Ref. [53]; ^2^ Ref. [54]; ^3^ Ref. [55].

## Appendix B. The Experimental Viscosity Data of Binary Resin Mixtures, Calculated Viscosities Based on Arrhenius Equation, and Binary Interaction Parameters

**Table A2:** The experimental viscosity data of binary resin mixtures, calculated viscosities based on Arrhenius equation, and binary interaction parameters.

Binary Mixture (Weight Ratio)	*η*_exp_ (cP)	*η*_Arrhenius_ (cP)	*G*_12_
Bis-GMA:Bis-EMA = 1:1	14000	28400	−2.83
Bis-GMA:Bis-EMA = 1:2	5000	9200	−2.68
Bis-GMA:Bis-EMA = 2:1	47400	86400	−2.75
Bis-GMA:HDDMA = 1:1	130	290	−3.68
Bis-GMA:HDDMA = 1:2	34	62	−3.74
Bis-GMA:HDDMA = 2:1	840	2080	−3.63
Bis-EMA:HDDMA = 1:1	31	32	−0.09
Bis-EMA:HDDMA = 2:1	70	73	−0.16
Bis-EMA:HDDMA = 3:1	121	120	0.03
Bis-GMA:IBMA = 1:1	620	255	4.21
Bis-GMA:IBMA = 1:2	80	61	1.81
Bis-GMA:IBMA = 2:1	3900	1640	3.49
Bis-EMA:IBMA = 1:1	52	33	2.1
Bis-EMA:IBMA = 2:1	113	72	1.82
Bis-EMA:IBMA = 3:1	200	116	2.23
